# Supplementary material for: Polyketide synthase gene clusters in the ericoid mycorrhizal fungus Oidiodendron maius: genome-wide prediction, expression profiling, and functional characterization of knockout mutants
Source: Front Plant Sci. 2026 Sep 16;17:1924430. doi: 10.3389/fpls.2026.1924430 (PMC13623903; doi:10.3389/fpls.2026.1924430)
Supplement: Supplementary file 1 [file SupplementaryFile1.zip › Supplementary Materials/Supplementary Folder 1.zip!/Oidma1_AssemblyScaffolds_Repeatmasked/knownclusterblast/cluster36/jgi.p_Oidma1_44455_mibig_hits.html]

| MIBiG Protein | Description | MIBiG Cluster | MiBiG Product | % ID | % Coverage | BLAST Score | E-value |
| --- | --- | --- | --- | --- | --- | --- | --- |
| AAS90107.1 | CypX | BGC0000006 | Aflatoxin\_biosynthetic\_gene\_cluster | 40 | 94.8374760994 | 318 | 1.7e-86 |
| AAS90036.1 | CypX | BGC0000008 | Aflatoxin\_biosynthetic\_gene\_cluster | 40 | 94.8374760994 | 317 | 2.8e-86 |
| AAS90014.1 | CypX | BGC0000007 | Aflatoxin\_biosynthetic\_gene\_cluster | 39 | 94.8374760994 | 314 | 1.8e-85 |
| BAE71332.1 | cytochrome\_P450\_monooxygenase | BGC0000004 | Aflatoxin\_biosynthetic\_gene\_cluster | 39 | 94.8374760994 | 312 | 9.1e-85 |
| AAS90082.1 | CypX | BGC0000010 | Aflatoxin\_biosynthetic\_gene\_cluster | 39 | 94.8374760994 | 311 | 1.6e-84 |
| AAZ95016.1 | cytochrome\_P450\_monooxygenase | BGC0000048 | Dothistromin\_biosynthetic\_gene\_cluster | 36 | 95.0286806883 | 305 | 1.1e-82 |
| AAS90062.1 | CypX | BGC0000009 | Aflatoxin\_biosynthetic\_gene\_cluster | 38 | 93.690248566 | 305 | 1.5e-82 |
| ACH72911.1 | AflV | BGC0000011 | Aflatoxin/sterigmatocystin\_biosynthetic\_gene\_cluster | 38 | 92.7342256214 | 289 | 6.3e-78 |
| ALA62324.1 | methylphloroacetophenone\_synthase | BGC0001309\_c2 | Usnic\_acid\_biosynthetic\_gene\_cluster | 43 | 76.4818355641 | 284 | 3.5e-76 |
| AAC49192.1 | putative\_p450\_monooxygenase | BGC0000152 | Sterigmatocystin\_biosynthetic\_gene\_cluster | 51 | 40.1529636711 | 187 | 4.4e-47 |
| BAU61566.1 | putative\_cytochrome\_P450 | BGC0001375 | Penitrem\_biosynthetic\_gene\_cluster | 27 | 39.0057361377 | 57 | 4e-08 |
| ACB12553.1 | Fum6 | BGC0000063 | Fumonisin\_biosynthetic\_gene\_cluster | 29 | 38.240917782 | 51 | 3.8e-06 |
